# Supplementary material for: Multiple clades of regulators contribute to bacterial phosphate homeostasis and Staphylococcus aureus pathogenesis
Source: mBio. 2026 Apr 22;17(5):e00203-26. doi: 10.1128/mbio.00203-26 (PMC13170248; doi:10.1128/mbio.00203-26)
Supplement: Supplemental material — Supplemental figures, tables, and data set caption. [file mbio.00203-26-s0002.pdf]

Supplementary Materials for

Multiple clades of regulators contribute to bacterial phosphate homeostasis and *Staphylococcus aureus* pathogenesis

Caroline Vermilya *et al.*

*Corresponding author. Email: thomas-kehl-fie@uiowa.edu

This PDF file includes:

Figs. S1 to S10

Tables S1 to S4

Data S1

Supplementary Figures

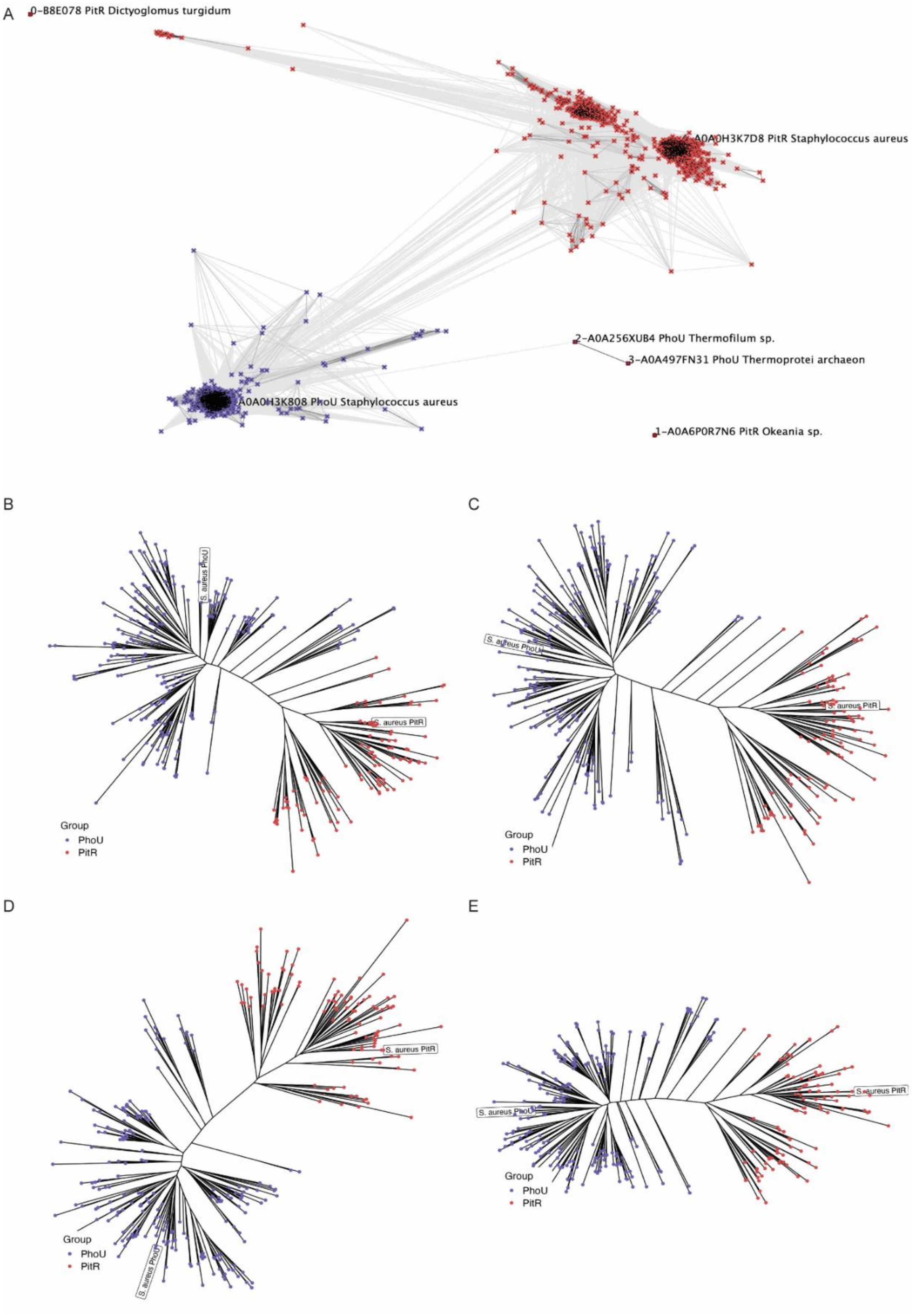

Supplemental Figure 1. CLANS force-directed graph based on sequence similarity reveals separate clusters of PhoU- and PitR-related proteins and gene tree result is robust to sampling and tree reconstruction parameters.

(A) Bacterial protein sequences related to the *S. aureus* PhoU (NWMN_1296) and PitR (NWMN_0632) were first clustered at the threshold of 0.7 sequence identity and then grouped at the genus level. One representative sequence per genus was retained, resulting in 2208 and 1226 sequences for each group, respectively. These sequences were subjected to the CLANS tool on the MPI Bioinformatics Toolkit server for all-against-all BLAST analysis. The results were visualized using the CLANS java program with the option of {cooling: 0.8; attraction: 10; attraction exponent: 1; repulsion: 10; repulsion exponent: 1}. Dots were colored based on the group (PhoU/PitR). *S. aureus* PhoU and PitR were labeled, as well as selected outliers. The color of the edge between nodes reflects the similarity in sequence, with darker gray being more similar. (B) As in Figure 1, PhoU- and PitR-related sequences were obtained from the InterPro database (Materials and Methods). After filtering by sequence identity at 70% and taxonomy at the genus level, 200 and 100 protein sequences were randomly chosen among the filtered representative sequences from each genus for both groups. Protein sequences were aligned using MAFFT v7 and a phylogenetic tree was reconstructed using RAxML v8.2.12 with LG+G substitution model with Gamma model for rate heterogeneity (-m PROTGAMMALG). (C) Tree based on the same random subset of sequences as in (B) but reconstructed utilizing the Dayhoff substitution matrix without rate heterogeneity (-m PROTCATDAYHOFF). (D) Tree based on a second random subset of sequences different from those utilized in (B-C), reconstructed as in (B). (E) Tree based on a third random subset of sequences different from those utilized in (B-D), reconstructed as in (B). Branch

lengths in the unrooted trees (B-E) are proportional to the sequence divergence. *S. aureus* PhoU and PitR sequences are labeled.

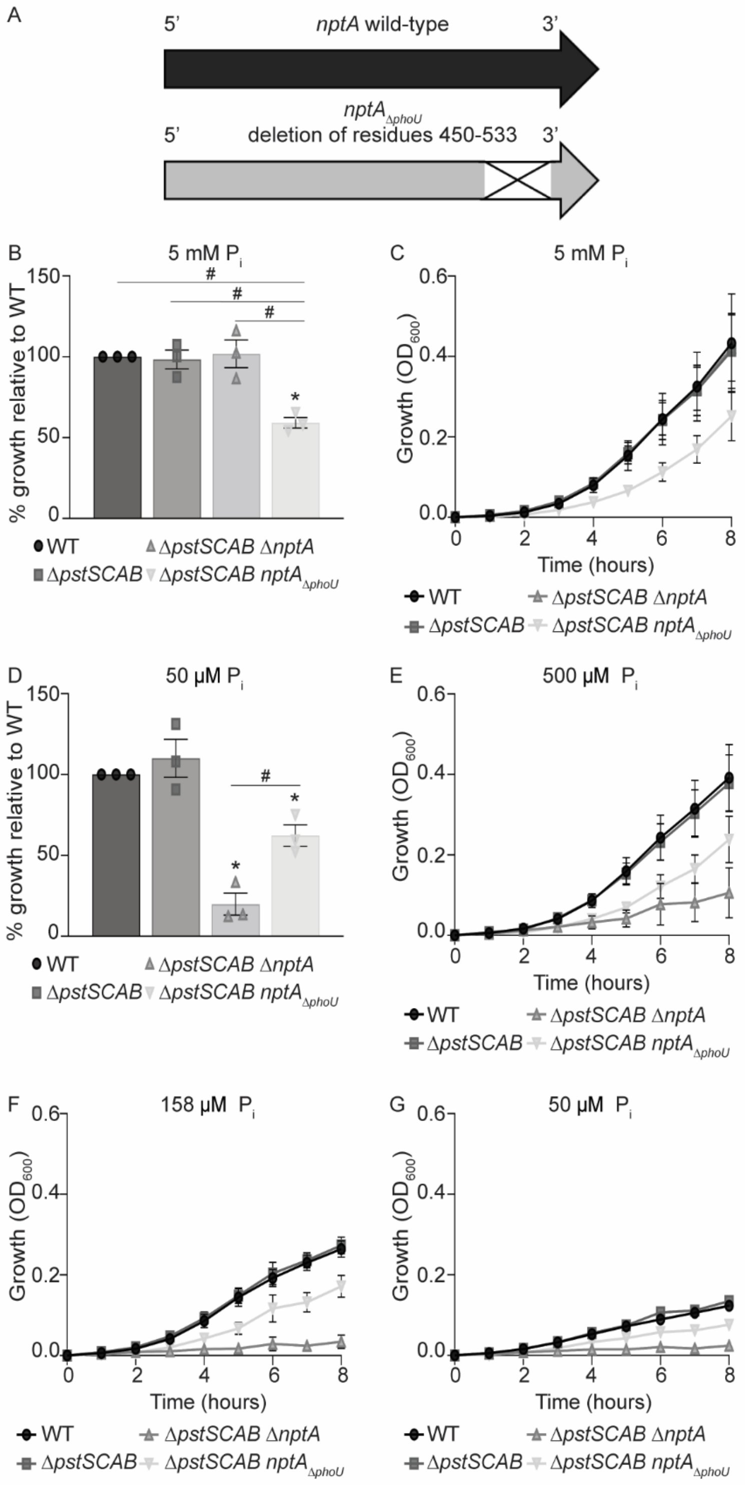

A
5’
nptA wild-type
3’
nptA∆phoU
5’
deletion of residues 450-533
3’
B
5 mM Pi
% growth relative to WT
150
100
50
0
#
#
#
*
WT
∆pstSCAB
∆pstSCAB ∆nptA
∆pstSCAB nptA∆phoU
C
5 mM Pi
Growth (OD600)
0.6
0.4
0.2
0.0
0
2
4
6
8
Time (hours)
WT
∆pstSCAB
∆pstSCAB ∆nptA
∆pstSCAB nptA∆phoU
D
50 µM Pi
% growth relative to WT
150
100
50
0
#
*
*
WT
∆pstSCAB
∆pstSCAB ∆nptA
∆pstSCAB nptA∆phoU
E
500 µM Pi
Growth (OD600)
0.6
0.4
0.2
0.0
0
2
4
6
8
Time (hours)
WT
∆pstSCAB
∆pstSCAB ∆nptA
∆pstSCAB nptA∆phoU
F
158 µM Pi
Growth (OD600)
0.6
0.4
0.2
0.0
0
2
4
6
8
Time (hours)
WT
∆pstSCAB
∆pstSCAB ∆nptA
∆pstSCAB nptA∆phoU
G
50 µM Pi
Growth (OD600)
0.6
0.4
0.2
0.0
0
2
4
6
8
Time (hours)
WT
∆pstSCAB
∆pstSCAB ∆nptA
∆pstSCAB nptA∆phoU

Supplemental Figure 2. Removal of the PhoU-domain from NptA does not prevent phosphate import.

(A) Diagram depicting wild-type *nptA* and $nptA_{\Delta phoU}$ PhoU-domain deletion construct. (B-G) *S. aureus* wild-type, Δ*pstSCAB*, Δ*pstSCAB*Δ*nptA*, and $\Delta pstSCABnptA_{\Delta phoU}$ were grown in PFM9, pH 8.4, supplemented with the indicated concentration of P_i. Growth was assessed by measuring absorbance at OD_{600}. $n \geq 3$. Error bars = SEM. (B & D) Growth relative to wild-type at 8 h. * = $P \leq 0.05$ relative to wild-type via one-way ANOVA with Tukey's posttest. # = $P \leq 0.05$ for the indicated comparison via one-way ANOVA with Tukey's posttest.

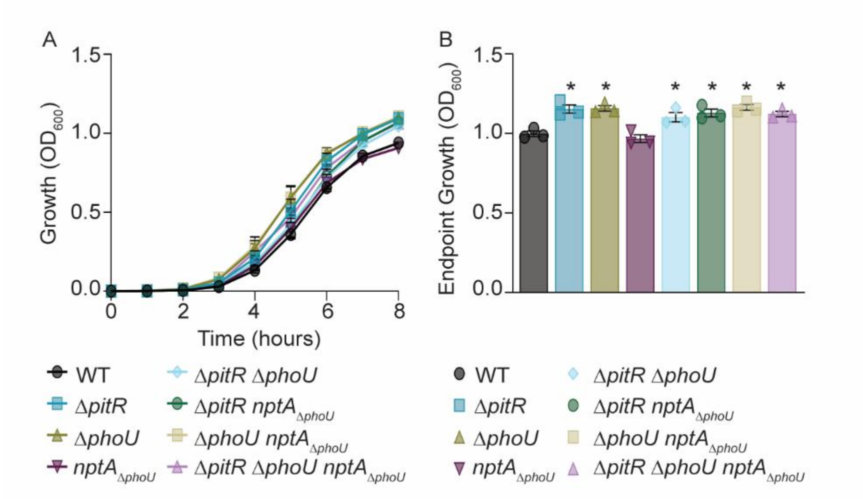

Supplemental Figure 3. Loss of all the PhoU homologs has a minimal impact on *S. aureus* growth in rich medium.

(A & B) *S. aureus* wild-type and the indicated strains were grown in TSB with growth assessed by measuring absorbance at OD_{600}. (B) Relative growth of the indicated strains at 6 h. Error bars = SEM. * = $P \leq 0.05$ relative to wild-type via one-way ANOVA with Tukey's posttest. (A & B) $n \geq 3$. Error bars = SEM.

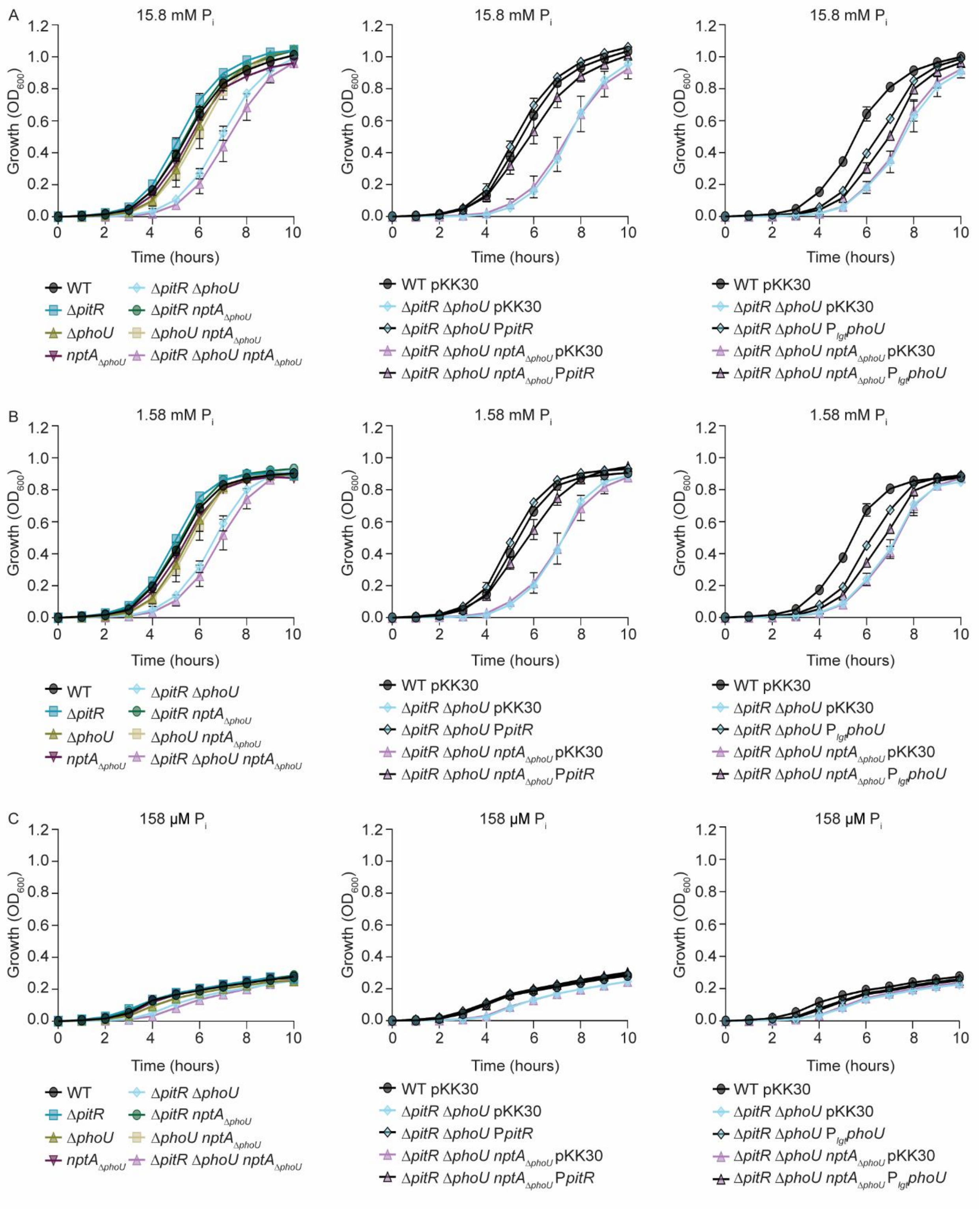
A
B
C
15.8 mM P_i
1.58 mM P_i
158 µM P_i
Growth (OD_{600})
Time (hours)
WT
ΔpitR
ΔphoU
nptA$_{\Delta phoU}$
ΔpitR ΔphoU
ΔpitR nptA$_{\Delta phoU}$
ΔphoU nptA$_{\Delta phoU}$
ΔpitR ΔphoU nptA$_{\Delta phoU}$
WT pKK30
ΔpitR ΔphoU pKK30
ΔpitR ΔphoU PpitR
ΔpitR ΔphoU nptA$_{\Delta phoU}$ pKK30
ΔpitR ΔphoU nptA$_{\Delta phoU}$ PpitR
ΔpitR ΔphoU P_{lgt}phoU
ΔpitR ΔphoU nptA$_{\Delta phoU}$ P_{lgt}phoU

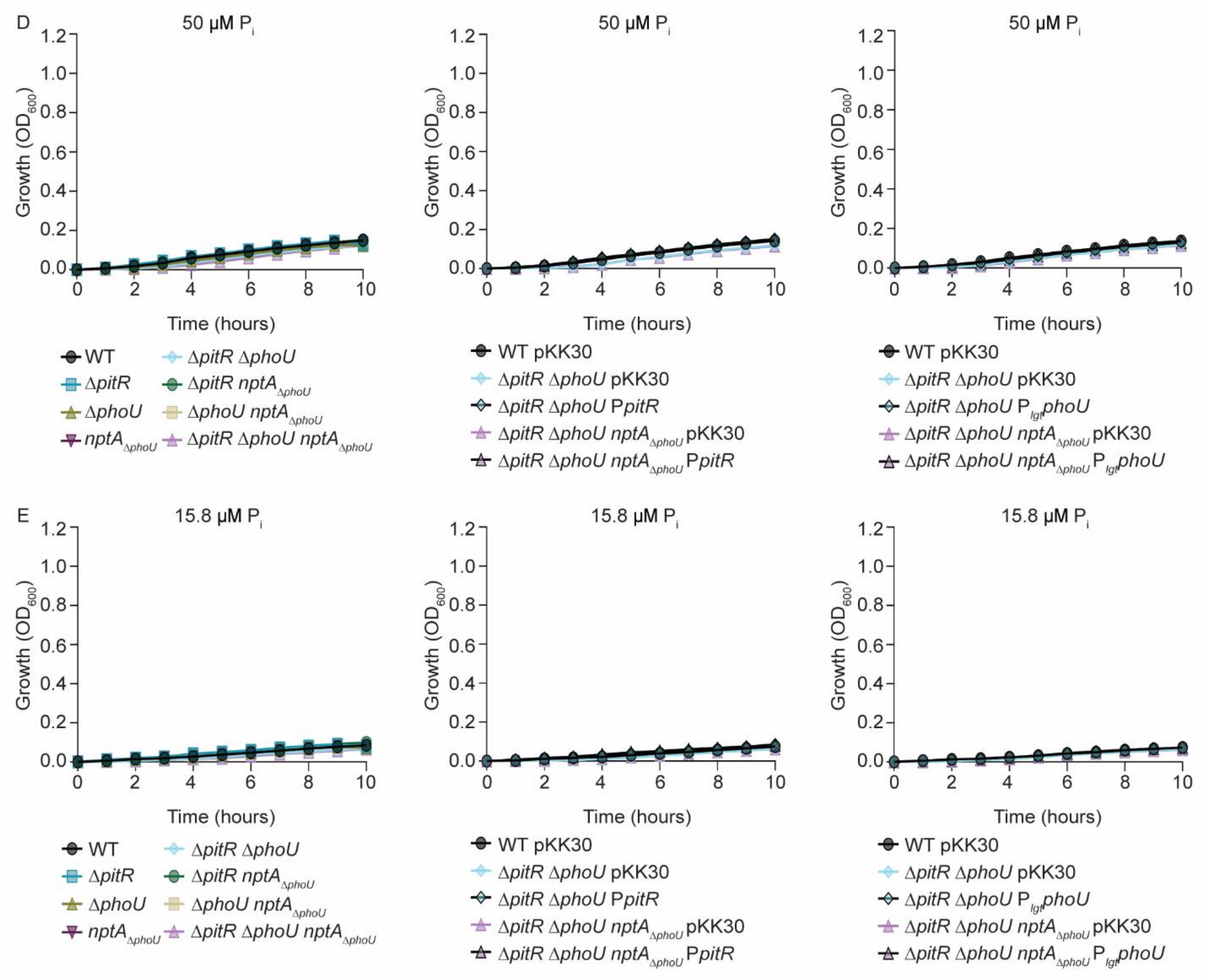

Supplemental Figure 4. The growth defects of strains lacking PitR and PhoU in a neutral medium can be complemented and are ablated as the P_i concentration decreases.

(A-E) *S. aureus* wild-type and the indicated strains were grown in PFM9 supplemented with the indicated P_i concentration at a pH of 7.4. Growth was assessed by measuring absorbance at OD_{600}. $n \geq 3$. Error bars = SEM. In the center column, the strains contain either an empty vector (pKK30) or a *pitR* complementation construct (P*pitR*) as indicated. In the right column, the strains contain either an empty vector (pKK30) or a *phoU* complementation construct (P_{lgt}*phoU*) as indicated.

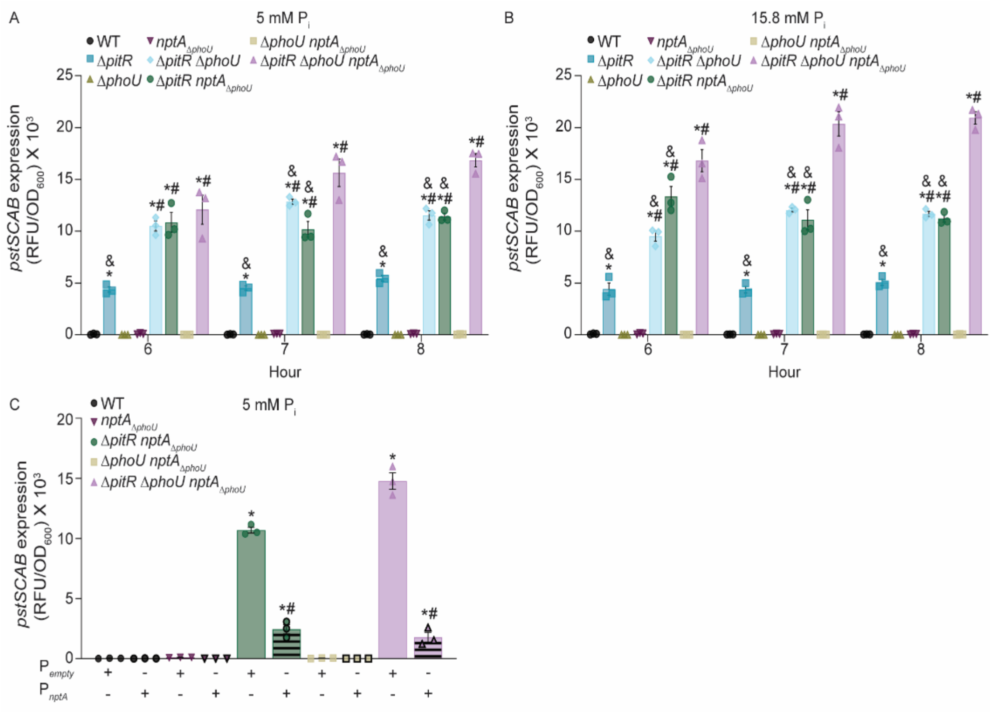

Supplemental Figure 5. The hierarchical expression pattern is consistent across multiple time points and the homeostasis defects associated with the loss of the PhoU-domain of NptA can be complemented.

(A & B) *S. aureus* wild-type and the indicated strains containing P*pstS-yfp* reporters were grown in PFM9, pH 7.4, supplemented with (A) 5 mM or (B) 15.8 mM P_i. The expression of *pstSCAB* was assessed by measuring fluorescence at T = 6, 7, and 8 h. * = $P \leq 0.05$ relative to wild-type at the same hour via one-way ANOVA with Tukey's posttest. # = $P \leq 0.05$ relative to Δ*pitR* at the same hour via one-way ANOVA with Tukey's posttest. & = $P \leq 0.05$ relative to Δ*pitR*Δ*phoUnptA*Δ*phoU* at the same hour via one-way ANOVA with Tukey's posttest. $n \geq 3$. Error bars = SEM. (C) *S. aureus* wild-type and the indicated strains containing P_{pstS}-*yfp* reporter and an empty vector (pKK30) or a *nptA* complementation construct (P*nptA*) were grown in PFM9, supplemented with 5 mM P_i. The expression of *pstSCAB* was assessed by measuring fluorescence at T = 8 h. * = $P \leq 0.05$ relative to wild-type at the same P_i concentration via one-way ANOVA

with Tukey's posttest. # = $P \leq 0.05$ relative to the parent strain carrying P_{empty} at the same P_i concentration via one-way ANOVA with Tukey's posttest. $n \geq 3$. Error bars = SEM.

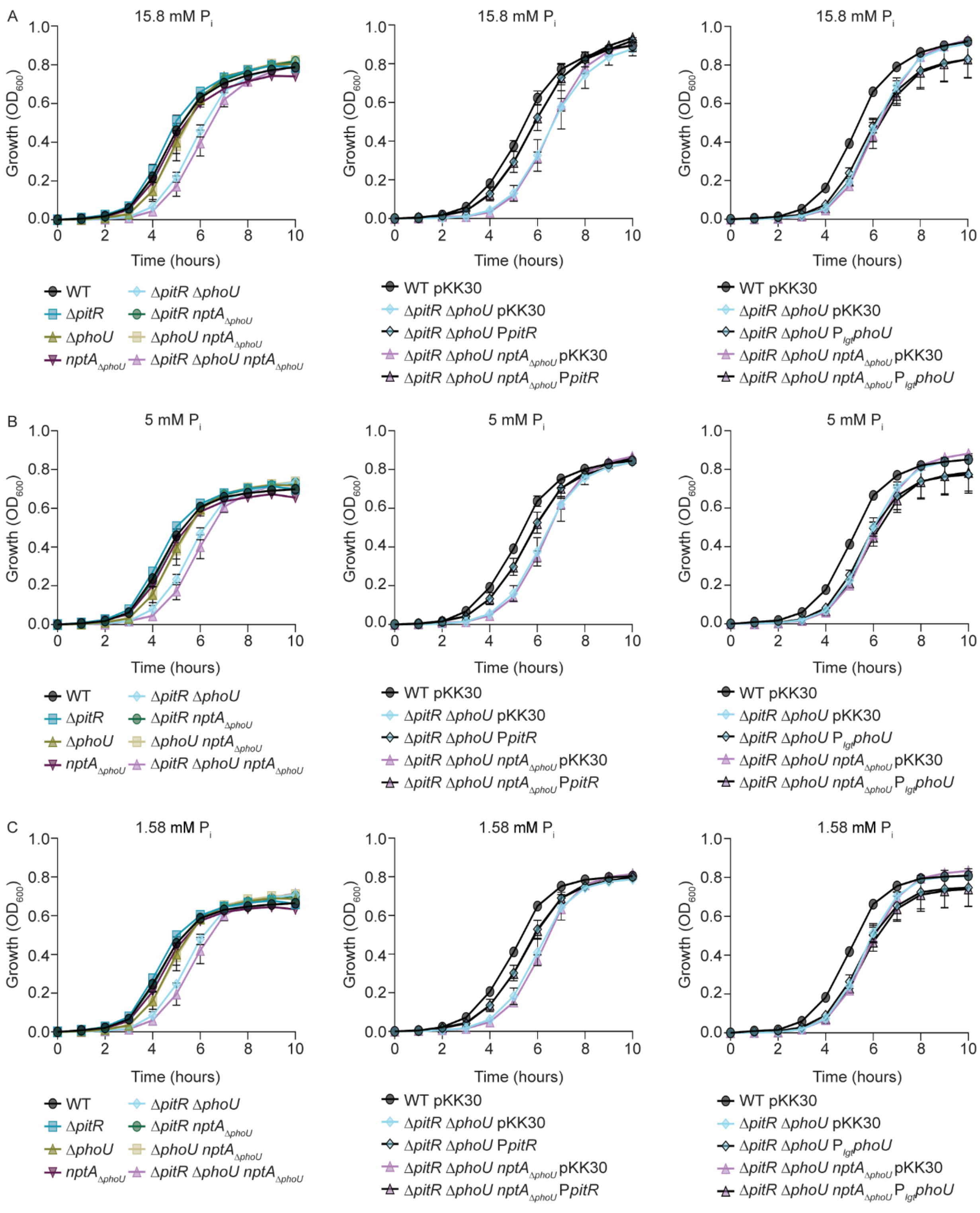
A
15.8 mM P$_i$
Growth (OD$_{600}$)
Time (hours)
WT
ΔpitR
ΔphoU
nptA$_{ΔphoU}$
ΔpitR ΔphoU
ΔpitR nptA$_{ΔphoU}$
ΔphoU nptA$_{ΔphoU}$
ΔpitR ΔphoU nptA$_{ΔphoU}$
WT pKK30
ΔpitR ΔphoU pKK30
ΔpitR ΔphoU PpitR
ΔpitR ΔphoU nptA$_{ΔphoU}$ pKK30
ΔpitR ΔphoU nptA$_{ΔphoU}$ PpitR
ΔpitR ΔphoU P$_{lgt}$phoU
ΔpitR ΔphoU nptA$_{ΔphoU}$ P$_{lgt}$phoU
B
5 mM P$_i$
C
1.58 mM P$_i$

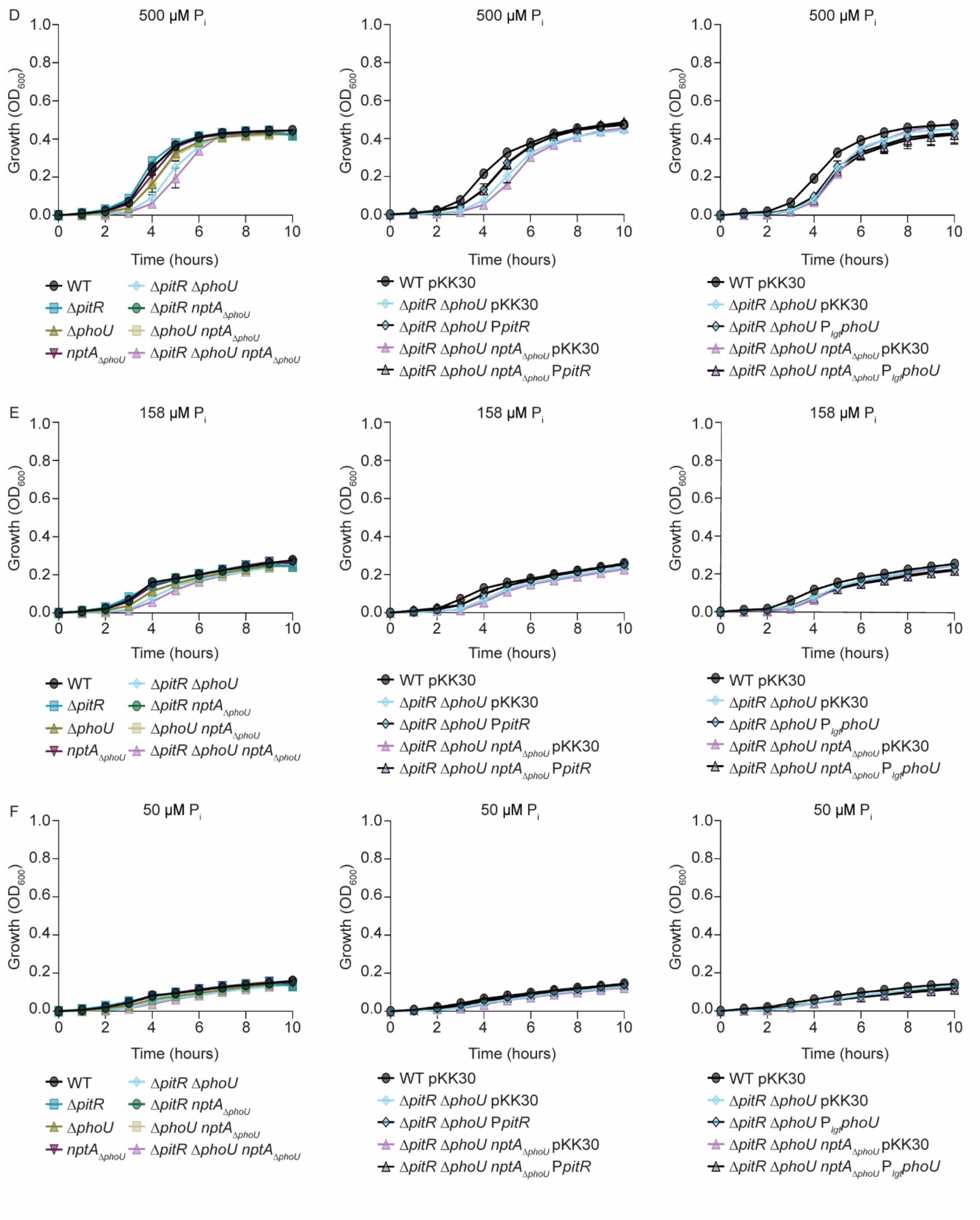

D
500 µM P_i
E
158 µM P_i
F
50 µM P_i
Growth (OD_{600})
Time (hours)
1.0
0.8
0.6
0.4
0.2
0.0
0
2
4
6
8
10
WT
ΔpitR
ΔphoU
$nptA_{\Delta phoU}$
ΔpitR ΔphoU
ΔpitR $nptA_{\Delta phoU}$
ΔphoU $nptA_{\Delta phoU}$
ΔpitR ΔphoU $nptA_{\Delta phoU}$
WT pKK30
ΔpitR ΔphoU pKK30
ΔpitR ΔphoU PpitR
ΔpitR ΔphoU $nptA_{\Delta phoU}$ pKK30
ΔpitR ΔphoU $nptA_{\Delta phoU}$ PpitR
ΔpitR ΔphoU P_{lgt}phoU
ΔpitR ΔphoU $nptA_{\Delta phoU}$ P_{lgt}phoU

Supplemental Figure 6. The growth defects of strains lacking PitR and PhoU in an acidic medium can be complemented and are ablated as the P_i concentration decreases. (A-F) *S. aureus* wild-type and the indicated strains were grown in PFM9 supplemented with the indicated P_i concentration at a pH of 6.4. Growth was assessed by measuring absorbance at OD_{600}. $n \geq 3$. Error bars = SEM. In the center column, the strains contain either an empty vector (pKK30) or a *pitR* complementation construct (P*pitR*) as indicated. In the right column, the strains contain either an empty vector (pKK30) or a *phoU* complementation construct (P_{lgt}*phoU*) as indicated.

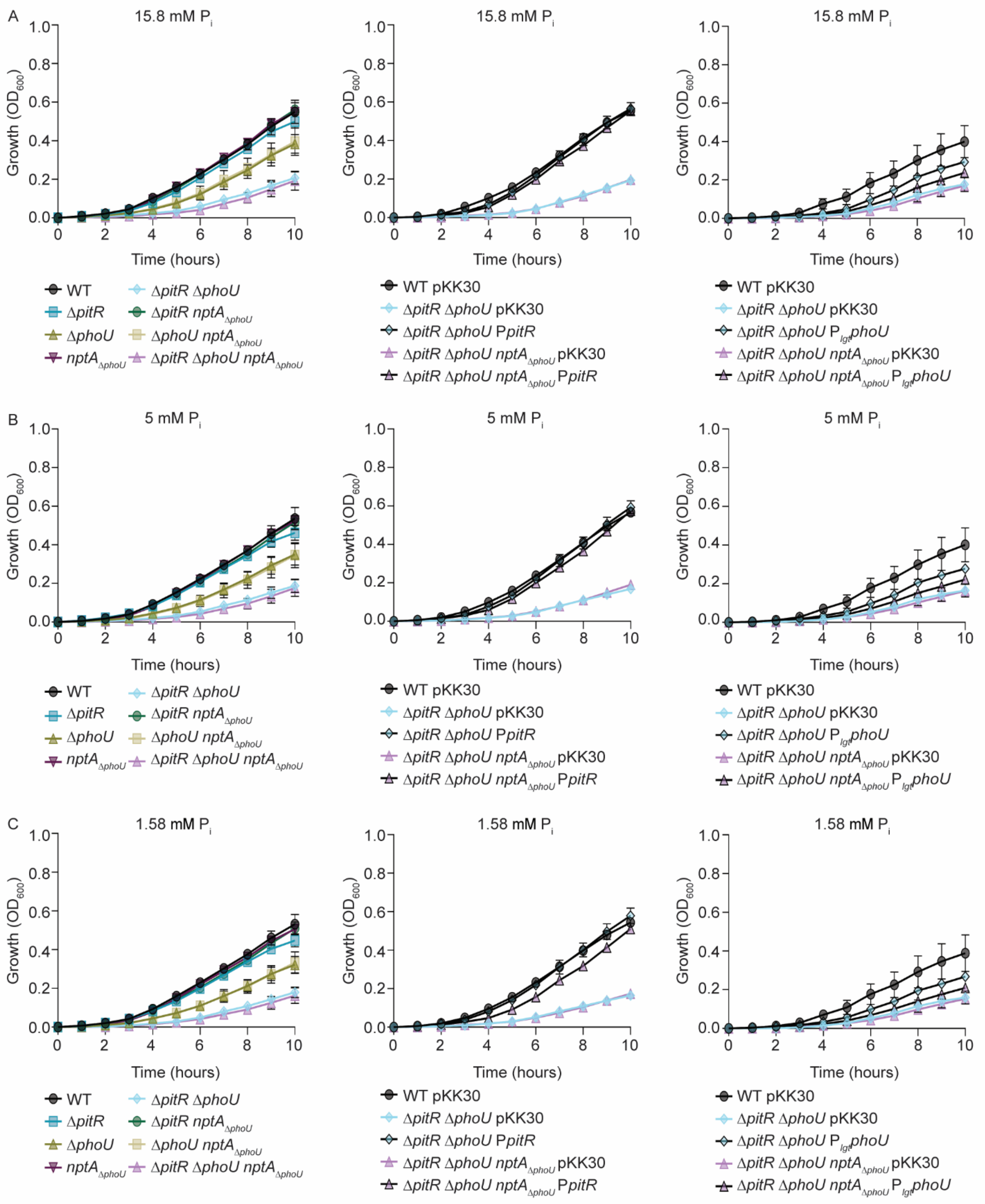
A
B
C
15.8 mM P_i
5 mM P_i
1.58 mM P_i
Growth (OD_{600})
Time (hours)
WT
ΔpitR
ΔphoU
$nptA_{\Delta phoU}$
ΔpitR ΔphoU
ΔpitR $nptA_{\Delta phoU}$
ΔphoU $nptA_{\Delta phoU}$
ΔpitR ΔphoU $nptA_{\Delta phoU}$
WT pKK30
ΔpitR ΔphoU pKK30
ΔpitR ΔphoU PpitR
ΔpitR ΔphoU $nptA_{\Delta phoU}$ pKK30
ΔpitR ΔphoU $nptA_{\Delta phoU}$ PpitR
ΔpitR ΔphoU $P_{lgt}phoU$
ΔpitR ΔphoU $nptA_{\Delta phoU}$ $P_{lgt}phoU$

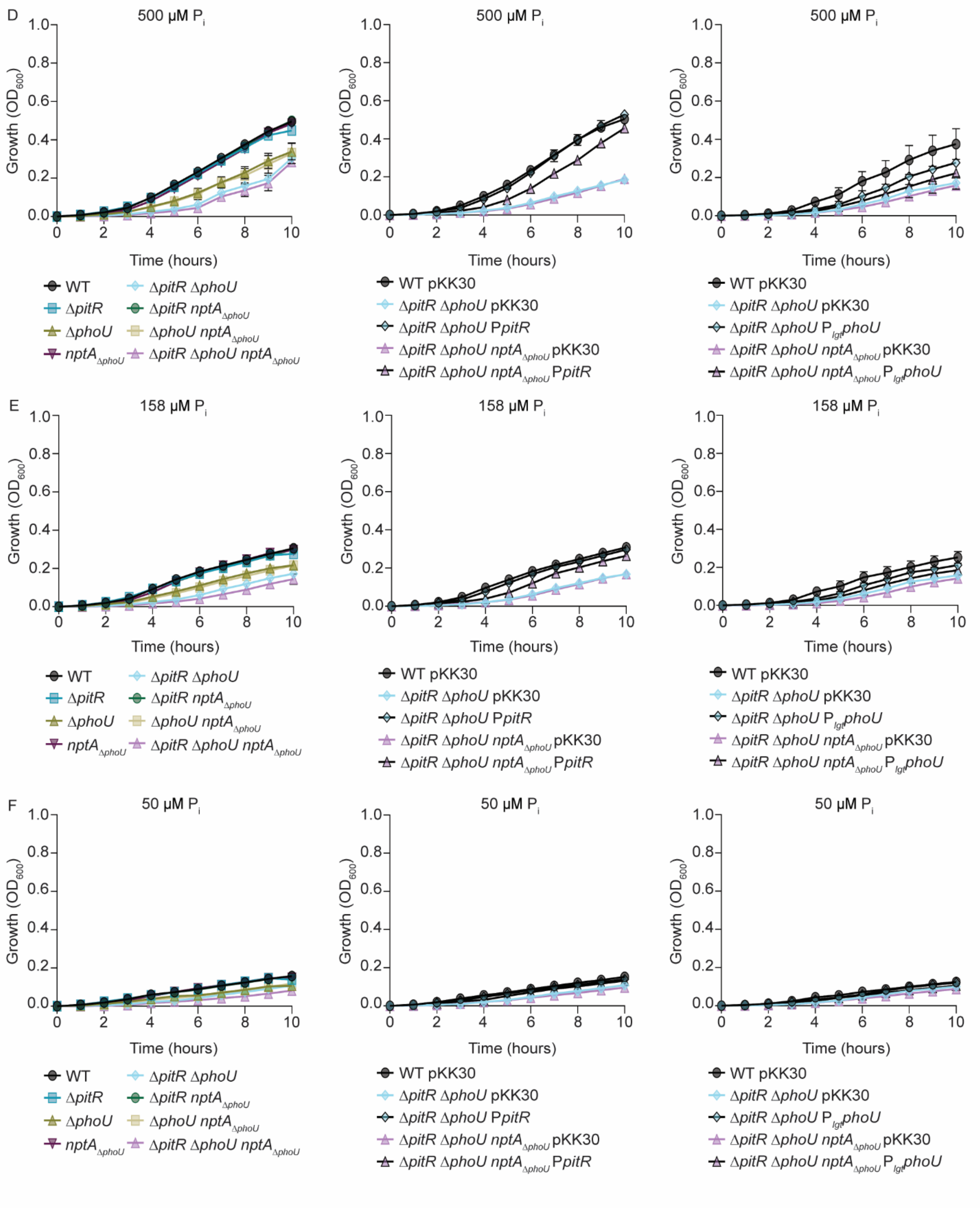
D
E
F
500 μM Pi
158 μM Pi
50 μM Pi
Growth (OD600)
Time (hours)
0.0
0.2
0.4
0.6
0.8
1.0
0
2
4
6
8
10
WT
ΔpitR
ΔphoU
nptAΔphoU
ΔpitR ΔphoU
ΔpitR nptAΔphoU
ΔphoU nptAΔphoU
ΔpitR ΔphoU nptAΔphoU
WT pKK30
ΔpitR ΔphoU pKK30
ΔpitR ΔphoU PpitR
ΔpitR ΔphoU nptAΔphoU pKK30
ΔpitR ΔphoU nptAΔphoU PpitR
ΔpitR ΔphoU Plgt phoU
ΔpitR ΔphoU nptAΔphoU Plgt phoU

Supplemental Figure 7. The growth defects of strains lacking PitR and PhoU in an alkaline medium can be complemented and are ablated as the P_i concentration decreases.

(A-F) *S. aureus* wild-type and the indicated strains were grown in PFM9 supplemented with the indicated P_i concentration at a pH of 8.4. Growth was assessed by measuring absorbance at OD_{600}. $n \geq 3$. Error bars = SEM. In the center column, the strains contain either an empty vector (pKK30) or a *pitR* complementation construct (P*pitR*) as indicated. In the right column, the strains contain either an empty vector (pKK30) or a *phoU* complementation construct (P_{lgt}*phoU*) as indicated.

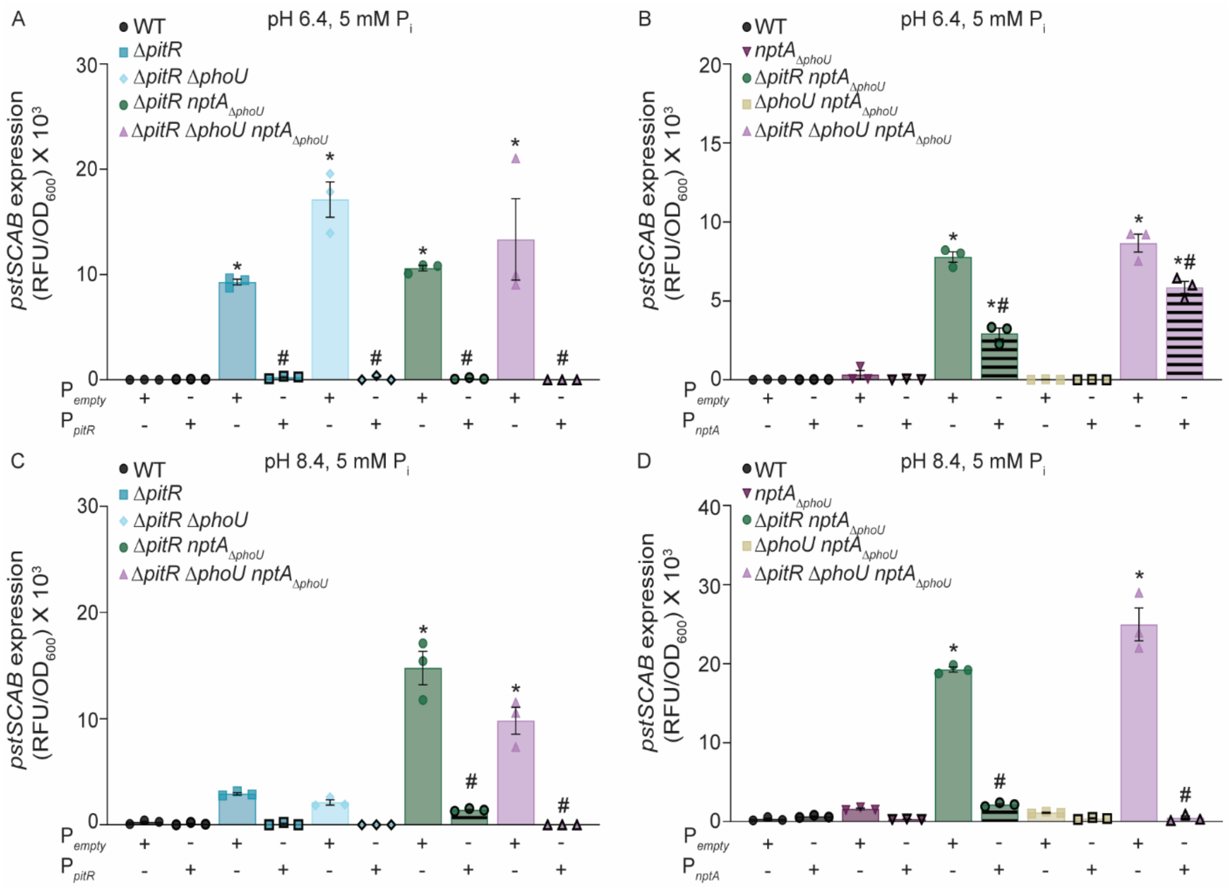

Supplemental Figure 8. The homeostasis defects associated with the loss of PitR or the PhoU-domain of NptA in acidic and alkaline media can be complemented.

(A-D) *S. aureus* wild-type and the indicated strains containing P_{pstS}-*yfp* reporter and an empty vector (pKK30), a *pitR* complementation construct (P*pitR*) (A & C), or *nptA* complementation construct (P*nptA*) (B & D) were grown in PFM9, supplemented with 5 mM, 500 µM, and 50 µM P_i at pH 6.4 (A & B) or pH 8.4 (C & D). The expression of *pstSCAB* was assessed by measuring fluorescence at T = 8 h. * = $P \leq 0.05$ relative to wild-type at the same P_i concentration via one-way ANOVA with Tukey's posttest. # = $P \leq 0.05$ relative to the parent strain carrying P_{empty} at the same P_i concentration via one-way ANOVA with Tukey's posttest. $n \geq 3$. Error bars = SEM.

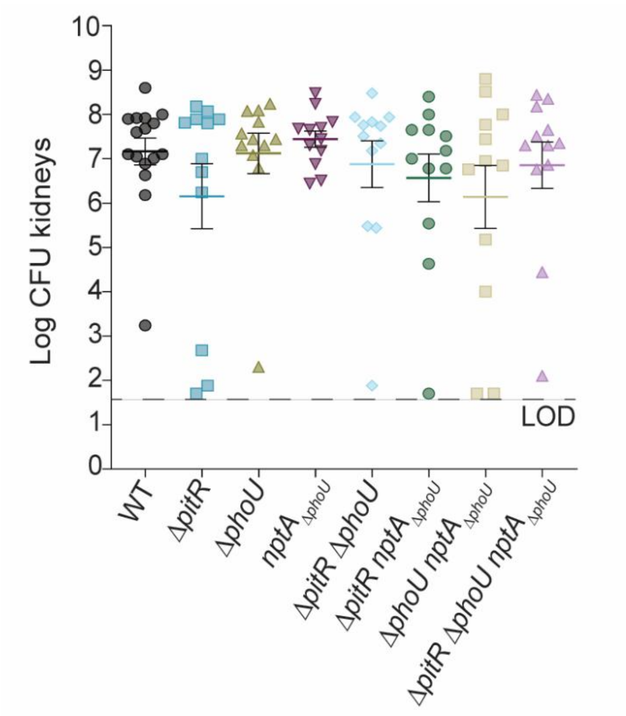

Supplemental Figure 9. Loss of the accessory regulators does not reduce bacterial burdens in the kidneys.

Wild-type C57BL/6J mice were systemically infected with *S. aureus* wild-type and the indicated strains and bacterial burdens in the kidneys were enumerated 4 days post-infection by plating for colony forming units. Lines indicate medians. The data are results from two independent experiments. $n \geq 15$ for each group.

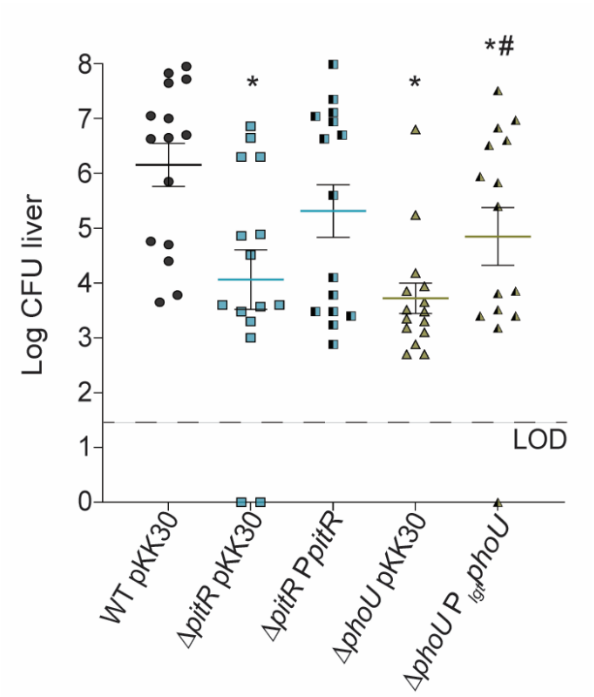

Supplemental Figure 10. The infection defects associated with loss of PitR and PhoU can be complemented.

Wild-type C57BL/6J mice were systemically infected with *S. aureus* wild-type and the indicated strains and bacterial burdens in the liver were enumerated 4 days post-infection by plating for colony forming units. * = $P < 0.05$ relative to wild-type pKK30 by Mann-Whitney test. # = $P < 0.05$ relative to Δ*phoU* pKK30 by Mann-Whitney test. Only significant *P* values are shown. Lines indicate medians. The data are results from two independent experiments. $n = 15$ for each group.

Supplementary Tables

Supplemental Table 1. Proteins used for the gene distribution analyses in this paper.

Abbrev.	Frac[a]	Annotation[b]
PhoR	0.744	Phosphate regulon sensor protein PhoR (SphS) (EC 2.7.13.3)
PhoP	0.753	Phosphate regulon transcriptional response regulator PhoP (=PhoB)
PhoU	0.780	Phosphate transport system regulatory protein PhoU
PstS	0.821	Phosphate ABC-transporter, substrate-binding protein PstS
PstS.m	0.010	Phosphate ABC-transporter, substrate-binding protein PstS, *Mycoplasma* type [c]
PstC	0.828	Phosphate ABC-transporter, permease protein PstC
PstA	0.829	Phosphate ABC-transporter, permease protein PstA
PstB	0.829	Phosphate ABC-transporter, ATP-binding protein PstB
PitA	0.428	Sodium-dependent phosphate transporter PitA
PitR	0.388	PhoU-like phosphate transport regulator PitR
SCO1846	0.049	Uncharacterized membrane protein SCO1846 [d]
NptA	0.279	Sodium-dependent phosphate transporter NptA [e]
NptA.v	0.041	Sodium-dependent phosphate transporter NptA, *Vibrio cholerae* type
NptA-PhoU	0.042	Sodium-dependent phosphate transporter NptA, without PhoU-domain

[a] Fraction of the 926 bacterial genomes surveyed that include the given protein annotation. The genomes and their annotation count data are in Table S1.

[b] Annotations are from a hand-curated database. The names are intended to be easily understandable, but do not match NCBI standards.

[c] These proteins are diverged in sequence; they were identified primarily by genomic context.

[d] SCO1846 is a locus tag from the original *Streptomyces coelicolor* A3(2) genome sequence. It corresponds to the NCBI nonredundant protein accession number WP_003976975 (hypothetical protein).

[e] This annotation is reserved for NptA proteins that include a PhoU-like domain.

Supplemental Table 2. ***Staphylococcus aureus*** **strains used in this study.**

Bacterial strain	Genotype	Reference
Newman WT	Wild-type	37
Newman Δ*pitR*	Δ*pitR*	This study
Newman Δ*phoU*	*phoU::erm*	37
Newman $nptA_{\Delta phoU}$	$nptA_{\Delta phoU}$	This study
Newman Δ*pitR*Δ*phoU*	Δ*pitR phoU::erm*	This study
Newman Δ*pitR*$nptA_{\Delta phoU}$	Δ*pitR* $nptA_{\Delta phoU}$	This study
Newman Δ*phoU*$nptA_{\Delta phoU}$	*phoU::erm* $nptA_{\Delta phoU}$	This study
Newman Δ*pitR*Δ*phoU*$nptA_{\Delta phoU}$	Δ*pitR phoU::erm* $nptA_{\Delta phoU}$	This study
Newman WT pAH5	Wild-type carrying pAH5	37
Newman Δ*pitR* pAH5	Δ*pitR* carrying pAH5	This study
Newman Δ*phoU* pAH5	*phoU::erm* carrying pAH5	37
Newman $nptA_{\Delta phoU}$ pAH5	$nptA_{\Delta phoU}$ carrying pAH5	This study
Newman Δ*pitR*Δ*phoU* pAH5	Δ*pitR phoU::erm* carrying pAH5	This study
Newman Δ*pitR*$nptA_{\Delta phoU}$ pAH5	Δ*pitR* $nptA_{\Delta phoU}$ carrying pAH5	This study
Newman Δ*phoU*$nptA_{\Delta phoU}$ pAH5	*phoU::erm* $nptA_{\Delta phoU}$ carrying pAH5	This study
Newman Δ*pitR*Δ*phoU*$nptA_{\Delta phoU}$ pAH5	Δ*pitR phoU::erm* $nptA_{\Delta phoU}$ carrying pAH5	This study
Newman WT P_{pstS}-*yfp*	Wild-type carrying P_{pstS}-*yfp*	36
Newman Δ*pitR* P_{pstS}-*yfp*	Δ*pitR* carrying P_{pstS}-*yfp*	This study
Newman Δ*phoU* P_{pstS}-*yfp*	*phoU::erm* carrying P_{pstS}-*yfp*	37
Newman $nptA_{\Delta phoU}$ P_{pstS}-*yfp*	$nptA_{\Delta phoU}$ carrying P_{pstS}-*yfp*	This study
Newman Δ*pitR*Δ*phoU* P_{pstS}-*yfp*	Δ*pitR phoU::erm* carrying P_{pstS}-*yfp*	This study
Newman Δ*pitR*$nptA_{\Delta phoU}$ P_{pstS}-*yfp*	Δ*pitR* $nptA_{\Delta phoU}$ carrying P_{pstS}-*yfp*	This study
Newman Δ*phoU*$nptA_{\Delta phoU}$ P_{pstS}-*yfp*	*phoU::erm* $nptA_{\Delta phoU}$ carrying P_{pstS}-*yfp*	This study
Newman Δ*pitR*Δ*phoU*$nptA_{\Delta phoU}$ P_{pstS}-*yfp*	Δ*pitR phoU::erm* $nptA_{\Delta phoU}$ carrying P_{pstS}-*yfp*	This study
Newman WT P_{nptA}-*yfp*	Wild-type carrying P_{nptA}-*yfp*	36
Newman Δ*pitR* P_{nptA}-*yfp*	Δ*pitR* carrying P_{nptA}-*yfp*	This study
Newman Δ*phoU* P_{nptA}-*yfp*	*phoU::erm* carrying P_{nptA}-*yfp*	37
Newman $nptA_{\Delta phoU}$ P_{nptA}-*yfp*	$nptA_{\Delta phoU}$ carrying P_{nptA}-*yfp*	This study
Newman Δ*pitR*Δ*phoU* P_{nptA}-*yfp*	Δ*pitR phoU::erm* carrying P_{nptA}-*yfp*	This study
Newman Δ*pitR*$nptA_{\Delta phoU}$ P_{nptA}-*yfp*	Δ*pitR* $nptA_{\Delta phoU}$ carrying P_{nptA}-*yfp*	This study
Newman Δ*phoU*$nptA_{\Delta phoU}$ P_{nptA}-*yfp*	*phoU::erm* $nptA_{\Delta phoU}$ carrying P_{nptA}-*yfp*	This study
Newman Δ*pitR*Δ*phoU*$nptA_{\Delta phoU}$ P_{nptA}-*yfp*	Δ*pitR phoU::erm* $nptA_{\Delta phoU}$ carrying P_{nptA}-*yfp*	This study
Newman WT pKK30	Wild-type carrying pKK30	This study
Newman Δ*pitR* pKK30	Δ*pitR* carrying pKK30	This study
Newman Δ*phoU* pKK30	*phoU::erm* carrying pKK30	This study
Newman Δ*pitR*Δ*phoU* pKK30	Δ*pitR phoU::erm* carrying pKK30	This study
Newman Δ*pitR*$nptA_{\Delta phoU}$ pKK30	Δ*pitR* $nptA_{\Delta phoU}$ carrying pKK30	This study
Newman Δ*pitR*Δ*phoU*$nptA_{\Delta phoU}$ pKK30	Δ*pitR phoU::erm* $nptA_{\Delta phoU}$ carrying pKK30	This study

Newman Δ*pitR* P*pitR*	Δ*pitR* carrying P*pitR*	This study
Newman Δ*pitR*Δ*phoU* P*pitR*	Δ*pitR phoU::erm* carrying P*pitR*	This study
Newman Δ*pitRnptA*$_{\Delta phoU}$ P*pitR*	Δ*pitR nptA*$_{\Delta phoU}$ carrying P*pitR*	This study
Newman Δ*pitR*Δ*phoUnptA*$_{\Delta phoU}$ P*pitR*	Δ*pitR phoU::erm nptA*$_{\Delta phoU}$ carrying P*pitR*	This study
Newman Δ*phoU* P$_{plgt}$-*phoU*	*phoU::erm* carrying P$_{plgt}$-*phoU*	This study
Newman WT pAH5 pKK30	Wild-type carrying pAH5 and pKK30	This study
Newman Δ*pitR* pAH5 pKK30	Δ*pitR* carrying pAH5 and pKK30	This study
Newman Δ*phoU* pAH5 pKK30	*phoU::erm* carrying pAH5 and pKK30	This study
Newman *nptA*$_{\Delta phoU}$ pAH5 pKK30	*nptA*$_{\Delta phoU}$ carrying pAH5 and pKK30	This study
Newman Δ*pitR*Δ*phoU* pAH5 pKK30	Δ*pitR phoU::erm* carrying pAH5 and pKK30	This study
Newman Δ*pitRnptA*$_{\Delta phoU}$ pAH5 pKK30	Δ*pitR nptA*$_{\Delta phoU}$ carrying pAH5 and pKK30	This study
Newman Δ*phoUnptA*$_{\Delta phoU}$ pAH5 pKK30	*phoU::erm nptA*$_{\Delta phoU}$ carrying pAH5 and pKK30	This study
Newman Δ*pitR*Δ*phoUnptA*$_{\Delta phoU}$ pAH5 pKK30	Δ*pitR phoU::erm nptA*$_{\Delta phoU}$ carrying pAH5 and pKK30	This study
Newman WT P$_{pstS}$-*yfp* pKK30	Wild-type carrying P$_{pstS}$-*yfp* and pKK30	This study
Newman Δ*pitR* P$_{pstS}$-*yfp* pKK30	Δ*pitR* carrying P$_{pstS}$-*yfp* and pKK30	This study
Newman Δ*phoU* P$_{pstS}$-*yfp* pKK30	*phoU::erm* carrying P$_{pstS}$-*yfp* and pKK30	This study
Newman *nptA*$_{\Delta phoU}$ P$_{pstS}$-*yfp* pKK30	*nptA*$_{\Delta phoU}$ carrying P$_{pstS}$-*yfp* and pKK30	This study
Newman Δ*pitR*Δ*phoU* P$_{pstS}$-*yfp* pKK30	Δ*pitR phoU::erm* carrying P$_{pstS}$-*yfp* and pKK30	This study
Newman Δ*pitRnptA*$_{\Delta phoU}$ P$_{pstS}$-*yfp* pKK30	Δ*pitR nptA*$_{\Delta phoU}$ carrying P$_{pstS}$-*yfp* and pKK30	This study
Newman Δ*phoUnptA*$_{\Delta phoU}$ P$_{pstS}$-*yfp* pKK30	*phoU::erm nptA*$_{\Delta phoU}$ carrying P$_{pstS}$-*yfp* and pKK30	This study
Newman Δ*pitR*Δ*phoUnptA*$_{\Delta phoU}$ P$_{pstS}$-*yfp* pKK30	Δ*pitR phoU::erm nptA*$_{\Delta phoU}$ carrying P$_{pstS}$-*yfp* and pKK30	This study
Newman WT P$_{nptA}$-*yfp* pKK30	Wild-type carrying P$_{nptA}$-*yfp* and pKK30	This study
Newman Δ*pitR* P$_{nptA}$-*yfp* pKK30	Δ*pitR* carrying P$_{nptA}$-*yfp* and pKK30	This study
Newman Δ*phoU* P$_{nptA}$-*yfp* pKK30	*phoU::erm* carrying P$_{nptA}$-*yfp* and pKK30	This study
Newman *nptA*$_{\Delta phoU}$ P$_{nptA}$-*yfp* pKK30	*nptA*$_{\Delta phoU}$ carrying P$_{nptA}$-*yfp* and pKK30	This study
Newman Δ*pitR*Δ*phoU* P$_{nptA}$-*yfp* pKK3	Δ*pitR phoU::erm* carrying P$_{nptA}$-*yfp* and pKK30	This study
Newman Δ*pitRnptA*$_{\Delta phoU}$ P$_{nptA}$-*yfp* pKK30	Δ*pitR nptA*$_{\Delta phoU}$ carrying P$_{nptA}$-*yfp* and pKK30	This study
Newman Δ*phoUnptA*$_{\Delta phoU}$ P$_{nptA}$-*yfp* pKK30	*phoU::erm nptA*$_{\Delta phoU}$ carrying P$_{nptA}$-*yfp* and pKK30	This study
Newman Δ*pitR*Δ*phoUnptA*$_{\Delta phoU}$ P$_{nptA}$-*yfp* pKK30	Δ*pitR phoU::erm nptA*$_{\Delta phoU}$ carrying P$_{pstS}$-*yfp* and pKK30	This study
Newman WT P$_{pstS}$-*yfp* P*pitR*	Wild-type carrying P$_{pstS}$-*yfp* and P*pitR*	This study

Newman Δ*pitR* P_{pstS}-*yfp* P*pitR*	Δ*pitR* carrying P_{pstS}-*yfp* and P*pitR*	This study
Newman Δ*pitR*Δ*phoU* P_{pstS}-*yfp* P*pitR*	Δ*pitR phoU::erm* carrying P_{pstS}-*yfp* and P*pitR*	This study
Newman Δ*pitRnptA*$_{\Delta phoU}$ P_{pstS}-*yfp* P*pitR*	Δ*pitR nptA*$_{\Delta phoU}$ carrying P_{pstS}-*yfp* and P*pitR*	This study
Newman Δ*pitR*Δ*phoUnptA*$_{\Delta phoU}$ P_{pstS}-*yfp* P*pitR*	Δ*pitR phoU::erm nptA*$_{\Delta phoU}$ carrying P_{pstS}-*yfp* and P*pitR*	This study
Newman WT P_{nptA}-*yfp* P*pitR*	Wild-type carrying P_{nptA}-*yfp* and P*pitR*	This study
Newman Δ*pitR* P_{nptA}-*yfp* P*pitR*	Δ*pitR* carrying P_{nptA}-*yfp* and P*pitR*	This study
Newman Δ*pitR*Δ*phoU* P_{nptA}-*yfp* P*pitR*	Δ*pitR phoU::erm* carrying P_{nptA}-*yfp* and P*pitR*	This study
Newman Δ*pitRnptA*$_{\Delta phoU}$ P_{nptA}-*yfp* P*pitR*	Δ*pitR nptA*$_{\Delta phoU}$ carrying P_{nptA}-*yfp* and P*pitR*	This study
Newman Δ*pitR*Δ*phoUnptA*$_{\Delta phoU}$ P_{nptA}-*yfp* P*pitR*	Δ*pitR phoU::erm nptA*$_{\Delta phoU}$ carrying P_{nptA}-*yfp* and P*pitR*	This study
Newman WT P_{pstS}-*yfp* P_{plgt}-*phoU*	Wild-type carrying P_{pstS}-*yfp* and P_{plgt}-*phoU*	This study
Newman Δ*pitR* P_{pstS}-*yfp* P_{plgt}-*phoU*	Δ*pitR* carrying P_{pstS}-*yfp* and P_{plgt}-*phoU*	This study
Newman Δ*phoU* P_{pstS}-*yfp* P_{plgt}-*phoU*	*phoU::erm* carrying P_{pstS}-*yfp* and P_{plgt}-*phoU*	This study
Newman Δ*pitR*Δ*phoU* P_{pstS}-*yfp* P_{plgt}-*phoU*	Δ*pitR phoU::erm* carrying P_{pstS}-*yfp* and P_{plgt}-*phoU*	This study
Newman Δ*pitRnptA*$_{\Delta phoU}$ P_{pstS}-*yfp* P_{plgt}-*phoU*	Δ*pitR nptA*$_{\Delta phoU}$ carrying P_{pstS}-*yfp* and P_{plgt}-*phoU*	This study
Newman Δ*phoUnptA*$_{\Delta phoU}$ P_{pstS}-*yfp* P_{plgt}-*phoU*	*phoU::erm nptA*$_{\Delta phoU}$ carrying P_{pstS}-*yfp* and P_{plgt}-*phoU*	This study
Newman Δ*pitR*Δ*phoUnptA*$_{\Delta phoU}$ P_{pstS}-*yfp* P_{plgt}-*phoU*	Δ*pitR phoU::erm nptA*$_{\Delta phoU}$ carrying P_{pstS}-*yfp* and P_{plgt}-*phoU*	This study
Newman WT P_{pstS}-*yfp* P*nptA*	Wild-type carrying P_{pstS}-*yfp* and P*nptA*	This study
Newman *nptA*$_{\Delta phoU}$ P_{pstS}-*yfp* P*nptA*	*nptA*$_{\Delta phoU}$ carrying P_{pstS}-*yfp* and P*nptA*	This study
Newman Δ*phoUnptA*$_{\Delta phoU}$ P_{pstS}-*yfp* P*nptA*	*phoU::erm nptA*$_{\Delta phoU}$ carrying P_{pstS}-*yfp* and P*nptA*	This study
Newman Δ*pitRnptA*$_{\Delta phoU}$ P_{pstS}-*yfp* P*nptA*	Δ*pitR nptA*$_{\Delta phoU}$ carrying P_{pstS}-*yfp* and P*nptA*	This study
Newman Δ*pitR*Δ*phoUnptA*$_{\Delta phoU}$ P_{pstS}-*yfp* P*nptA*	Δ*pitR phoU::erm nptA*$_{\Delta phoU}$ carrying P_{pstS}-*yfp* and P*nptA*	This study

159

Supplemental Table 3. Plasmids used in this study.

**Plasmid**	**Description**	**Reference**
pKOR1	Allelic replacement vector	88
pKOR1::*pitR*	*pitR* deletion allelic replacement vector	This study
pKOR1::*phoU*	*phoU* deletion allelic replacement vector	This study
pKOR1::$nptA_{\Delta phoU}$	*nptA* PhoU-domain removal allelic replacement vector	This study
pAH5	YFP transcriptional reporter empty vector	37
pP_{pstS}-*yfp*	pAH5E with *pst* promoter driving *yfp* expression	36
pP_{nptA}-*yfp*	pAH5E with *nptA* promoter driving *yfp* expression	36
pKK30	Complementation empty vector	36
P*pitR*	Complementation plasmid with *pitR* controlled by native promoter	This study
P_{p}*lgt-phoU*	Complementation plasmid with *phoU* controlled by the staphylococcal *lgt* promoter	This study
P*nptA*	Complementation plasmid with *nptA* controlled by native promoter	This study

Supplemental Table 4. Primers used in this study.

Primer	Sequence
phoU KO 5' F	AGCTATGACCATGTAATACGACTCACTATAGGGGATATCCAGAATGTTGCATCGGC
phoU KO 5' R	TTTAAGTTATTGTTCGTAAATTATTGCCATTATATATCAACCAAAC
phoU KO 3' F	TATATAATGGCAATAATTTACGAACAATAACTTAAAGTTATTACTATAAAATC
phoU KO 3' R	ACGACCGAGCGCAGCGAGTCAGTGAGCGAGGAGGAAGAGCTGTAGACCCTGATTATGAAC
pitR KO 5' F	AGCTATGACCATGTAATACGACTCACTATAGGGGATATCGATGAAACCGAAGATGAG
pitR KO 5' R	CTCCCCTTAGCTATTTTTCTTACTAAACATTTAAAATCCTCC
pitR KO 3' F	TTTTAAATGTTTAGTAAGAAAAATAGCTAAGGGGAGTATATATTTATG
pitR KO 3' R	ACCGAGCGCAGCGAGTCAGTGAGCGAGGAGGAAGAGCGAATAGTAAACCTGCTAACAATG
nptAphoU KO 5’ F	GACCATGTAATACGACTCACTATAGGGGATCAATTATAAGCGATAATCTAAATATAAAGC
nptAphoU KO 5’ R	GCCAACAAGTGCTTCACCGCGTTGTGCATCTTC
nptAphoU KO 3’ F	GATGCACAACGCGGTGAAGCACTTGTTGGCCTTAAC
nptAphoU KO 3’ R	GACCGAGCGCAGCGAGTCAGTGAGCGAGGAAGTTGGTGAAAAATTATTACAAAAAATG
plgt F	AGTGACAGGCGATGCGGCCGCTAGCGAATTTCTTTTCGAAATTCTTTATG
plgt R	TTATTGCCATGCATGCTAAAAAACGATTC
phoU comp F	TTTAGCATGCATGGCAATAATTAGACAACG
phoU comp R	GGATCCCCGGGTACCGAGCTCTTATTGTTCGTAATGTGTACC
pitR comp F	AGTGACAGGCGATGCGGCCGCTAGCAATATACAGATGGCTTTCAG
pitR comp R	TGCTTGTAATTCATGATTCGGATCCTTAGCTATTTTTCATAATAATAGTTTCAAAATTATTG
nptA comp F	AGCAAAGTGACAGGCGATGCTATTGCGTTGTTCTAAAAATTTGG
nptA comp R	TGCTTGTAATTCATGATTCGACGAAAACCATTAATAGATTTTTATTTG

Supplementary Data

Supplemental Data Set 1. Genomes and analysis of phosphate importers and regulators used in this manuscript
